# Targeting the Type II Secretion System: Development, Optimization, and Validation of a High-Throughput Screen for the Identification of Small Molecule Inhibitors

**DOI:** 10.3389/fcimb.2017.00380

**Published:** 2017-08-28

**Authors:** Ursula Waack, Tanya L. Johnson, Khalil Chedid, Chuanwu Xi, Lyle A. Simmons, Harry L. T. Mobley, Maria Sandkvist

**Affiliations:** ^1^Department of Microbiology and Immunology, University of Michigan Medical School Ann Arbor, MI, United States; ^2^Department of Chemistry, Eastern Michigan University Ypsilanti, MI, United States; ^3^Department of Environmental Health Sciences, University of Michigan School of Public Health Ann Arbor, MI, United States; ^4^Department of Molecular, Cellular, and Developmental Biology, University of Michigan Ann Arbor, MI, United States

**Keywords:** *Acinetobacter baumannii*, type II secretion, high-throughput screening, small molecule inhibitors, LipA, lipase activity, antibiotic resistance, antidepressants

## Abstract

Nosocomial pathogens that develop multidrug resistance present an increasing problem for healthcare facilities. Due to its rapid rise in antibiotic resistance, *Acinetobacter baumannii* is one of the most concerning gram-negative species. *A. baumannii* typically infects immune compromised individuals resulting in a variety of outcomes, including pneumonia and bacteremia. Using a murine model for bacteremia, we have previously shown that the type II secretion system (T2SS) contributes to *in vivo* fitness of *A. baumannii*. Here, we provide support for a role of the T2SS in protecting *A. baumannii* from human complement as deletion of the T2SS gene *gspD* resulted in a 100-fold reduction in surviving cells when incubated with human serum. This effect was abrogated in the absence of Factor B, a component of the alternative pathway of complement activation, indicating that the T2SS protects *A. baumannii* against the alternative complement pathway. Because inactivation of the T2SS results in loss of secretion of multiple enzymes, reduced *in vivo* fitness, and increased sensitivity to human complement, the T2SS may be a suitable target for therapeutic intervention. Accordingly, we developed and optimized a whole-cell high-throughput screening (HTS) assay based on secreted lipase activity to identify small molecule inhibitors of the T2SS. We tested the reproducibility of our assay using a 6,400-compound library. With small variation within controls and a dynamic range between positive and negative controls, the assay had a z-factor of 0.65, establishing its suitability for HTS. Our screen identified the lipase inhibitors Orlistat and Ebelactone B demonstrating the specificity of the assay. To eliminate inhibitors of lipase activity and lipase expression, two counter assays were developed and optimized. By implementing these assays, all seven tricyclic antidepressants present in the library were found to be inhibitors of the lipase, highlighting the potential of identifying alternative targets for approved pharmaceuticals. Although no T2SS inhibitor was identified among the compounds that reduced lipase activity by ≥30%, our small proof-of-concept pilot study indicates that the HTS regimen is simple, reproducible, and specific and that it can be used to screen larger libraries for the identification of T2SS inhibitors that may be developed into novel *A. baumannii* therapeutics.

## Introduction

A growing concern in hospitals, nursing homes, and other healthcare facilities is the increasing frequency of antibiotic resistant infections that result in longer hospital stays, higher costs, and increased mortality. The ESKAPE pathogens *Enterococcus faecium, Staphylococcus aureus, Klebsiella pneumoniae, Acinetobacter baumannii, Pseudomonas aeruginosa*, and *Enterobacter* species have attracted considerable attention as they cause the majority of nosocomial infections (Rice, 2008). Infections caused by *A. baumannii* are prevalent with ~45,000 cases per year in the United States alone. Globally, there are about 1 million cases annually (Spellberg and Rex, 2013) and reports suggest that *A. baumannii* may be the leading cause of nosocomial infections in some countries (Wong et al., 2016). It is estimated that 50% of these infections are caused by antibiotic-resistant strains (Spellberg and Rex, 2013). Exposure to *A. baumannii* can result in a variety of infections including pneumonia, urinary tract infection, bacteremia, meningitis, skin, and wound infections that may lead to sepsis (Bergogne-Berezin and Towner, 1996; Maragakis and Perl, 2008). Considered an opportunist, *A. baumannii* typically infects immune-compromised individuals but more recently isolated strains may not be restricted to this patient population, possibly as a consequence of increased virulence (Jones et al., 2015; Paterson and Harris, 2015). The remarkable ability of *A. baumannii* to form biofilm and resist dry environments (Jawad et al., 1998; Espinal et al., 2012) may explain its prevalence in healthcare environments (Weernink et al., 1995; Catalano et al., 1999). Additional contributing factors include multi- or pan-antibiotic resistance (Maragakis and Perl, 2008; Leite et al., 2016), which is due, in part, to intrinsic properties of the outer membrane of *A. baumannii* and its notable ability to acquire foreign DNA through horizontal gene transfer (de Vries and Wackernagel, 2002).

The rise in antibiotic resistance rapidly reduces the options of effective treatment and calls for the identification of new therapeutic approaches. A recommended strategy combines antibiotics with drugs that target resistance mechanisms such as Augmentin, which consists of Amoxicillin and the β-lactamase inhibitor Clavulanate. Other feasible options include the combination of antibiotics with inhibitors of drug efflux pumps or outer membrane permeabilizers (Gill et al., 2015). Identification of new therapeutic targets is also necessary. These may include essential processes such as lipopolysaccharide synthesis and transport as well as factors that contribute to *in vivo* fitness and virulence.

One of the first studies to target virulence factors using HTS of small molecule libraries identified a compound that inhibits dimerization of ToxT, a virulence regulator in *Vibrio cholerae* (Hung et al., 2005; Shakhnovich et al., 2007). This inhibitor abolishes the production of cholera toxin and decreases TCP-mediated colonization in an infant mouse model (Hung et al., 2005). Other studies have screened for biologicals or chemical compounds that target colonization factors, such as curli and type 1 pili, toxins, protein secretion pathways or quorum sensing systems (Steadman et al., 2014; Gill et al., 2015; Ruer et al., 2015; Hauser et al., 2016). With a few exceptions, it is too soon to evaluate the outcome of these studies and their success; however, some of these potential anti-virulence drugs are in various stages of development and are being analyzed in animal models or clinical trials (Pan et al., 2009; Rasko and Sperandio, 2010; Hauser et al., 2016). An IgG antibody that targets the binding of anthrax toxin to its receptor is currently used as an antitoxin in combination therapy for the treatment of *Bacillus anthracis* infections (Hendricks et al., 2014) and demonstrates the feasibility of targeting disease-causing components of pathogens.

**Figure 1:**
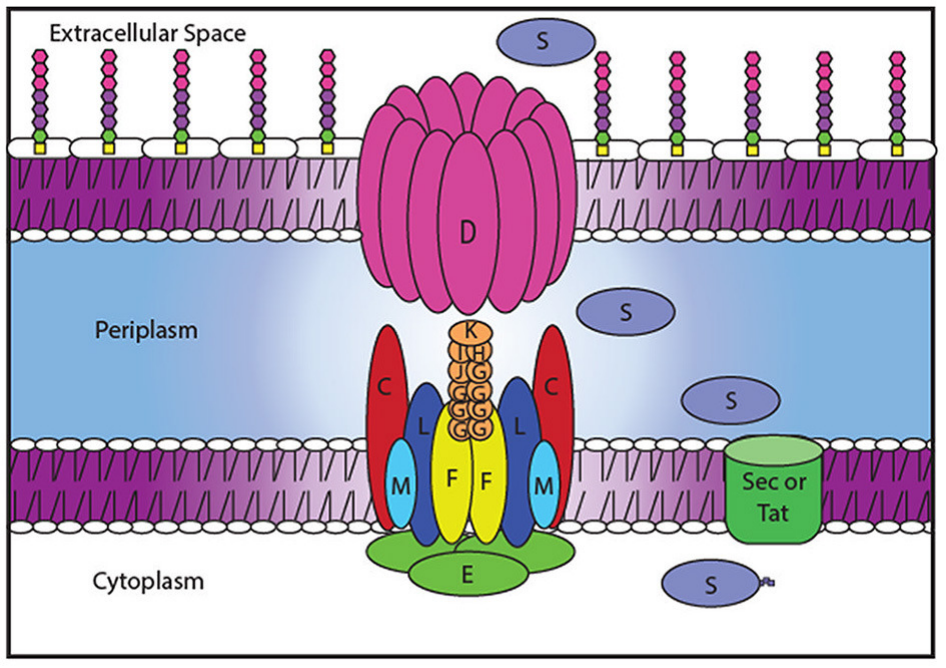
Potential Targets of a T2SS inhibitor. T2SS inhibitors may block transcription of the T2SS genes or translation, post-translational modification, protein-protein interactions or function of the T2SS proteins. Blocking the recognition by GspC/GspD of the T2SS substrates (S) to be secreted by the T2SS system could also halt secretion.

Secretion systems are particularly attractive targets for alternative therapeutics as their inactivation interferes with the delivery of entire batteries of secreted virulence factors. Therefore, several HTSs have been designed to identify small molecule inhibitors of the type III secretion system, which is present in many gram-negative human pathogens and secretes a wide variety of virulence effectors (Nordfelth et al., 2005; Aiello et al., 2010). Another secretion system, the type II secretion system (T2SS), is responsible for the secretion of numerous degradative enzymes and toxins that contribute to survival in the environment and the mammalian host and may also be a suitable target for alternative therapeutics (Sandkvist, 2001; Cianciotto, 2005; Cianciotto and White, 2017). As with many gram-negative pathogens, *A. baumannii* possesses a functional T2SS (Elhosseiny et al., 2016; Johnson et al., 2016). The T2SS forms an apparatus that spans both the inner and outer membrane and is encoded by 12 essential genes, *gspC-M* and *pilD* (Korotkov et al., 2012; Thomassin et al., 2017). T2S substrates are synthesized with an N-terminal signal peptide that allows for translocation from the cytoplasm to the periplasm via the general export (Sec) or twin arginine translocation (Tat) pathways. Once in the periplasm, the signal sequence is cleaved, the protein folds, and interacts with the T2SS to finally exit the cell via a gated outer membrane pore formed by GspD (Reichow et al., 2010; Douzi et al., 2011; Yan et al., 2017). GspD connects to GspC, one of the components of the inner membrane platform that also consists of the transmembrane proteins GpsF, L, and M (Sandkvist et al., 1999; Py et al., 2001; Michel et al., 2007; Abendroth et al., 2009; Douzi et al., 2011; Korotkov et al., 2011). The pseudopilins GspG, H, I, J, and K make a pseudopilus, a structure homologous to the Type IV pilus, while PilD cleaves and methylates the pseudopilin subunits prior to their assembly (Nunn and Lory, 1993; Durand et al., 2005; Cisneros et al., 2012). The entire system is powered by a cytoplasmic ATPase, GspE (Camberg and Sandkvist, 2005; Camberg et al., 2007). All of these proteins, including their expression and interactions, are potential targets for a therapeutic compound (Figure 1).

Recent work by our laboratory and others has demonstrated the benefit of having a functional T2SS for colonization by *A*. *baumannii* and *A. nosocomialis* (Elhosseiny et al., 2016; Harding et al., 2016; Johnson et al., 2016). Inactivation of the T2SS or one of its substrates results in diminished survival in murine models for bacteremia and pneumonia, indicating that screening for compounds that target the T2SS may lead to the identification of *A. baumannii* virulence inhibitors. In this study, we describe the development and optimization of a HTS to identify small molecule inhibitors of the T2SS in *A. baumannii*. In addition, we highlight the need for inclusion of high-throughput counter-screens to remove compounds with alternative targets.

## Methods

### Bacterial strains and plasmids

All bacterial strains and plasmids are described in Table 1. All strains were cultured overnight in Luria-Bertani (LB) broth. When necessary, LB broth was supplemented with carbenicillin (100 μg/ml) for plasmid maintenance. The studies were conducted under biosafety level 2 conditions.

**Table 1:** List of bacterial strains and plasmids used in study.

**Strain or plasmid**	**Characteristics**	**Source or study**
**STRAIN**		
17978	WT for this study	ATCC
17978Δ*gspD*	T2SS mutant	Johnson et al., 2016
17978Δ*gspN*		Johnson et al., 2016
17978Δ*lipA*		Johnson et al., 2016
AB031	Clinical strain	Tilley et al., 2014
AB031 Δ*gspD*	T2SS mutant	Waack et al., in preparation.
AB 0057	Clinical strain, tet^R^, chl^R^, trim^R^, carb^R^	Adams et al., 2008
AB 5075	Clinical strain, tet^R^, rif^R^, carb^R^, trim^R^	Jacobs et al., 2014
P020	Clinical strain, cef^R^	Greene et al., 2016
C038	Clinical strain, cef^R^	Greene et al., 2016
C058	Clinical strain, mer^R^, azt^R^, cef^R^, cip^R^, lev^R^, imi^R^	Greene et al., 2016
C076	Clinical strain, azt^R^, cef^R^, cip^R^, lev^R^, trim^R^	Greene et al., 2016
P084	Clinical strain, mer^R^, azt^R^, cef^R^, cip^R^, lev^R^, imi^R^	Greene et al., 2016
C097	Clinical strain	Greene et al., 2016
P102	Clinical strain, mer^R^, azt^R^, cef^R^, cip^R^, lev^R^, imi^R^	Greene et al., 2016
P143	Clinical strain, azt^R^, cef^R^	Greene et al., 2016
**PLASMID**		
pMMB67EH	Low copy vector (Ap^r^)	Fürste et al., 1986
p*lipBA*	pMMB67EH-*lipBA* (Ap^r^)	Johnson et al., 2016
p*gfp*	pMMB67EH-*gfp* (Ap^r^)	Scott et al., 2001

*azt, aztreonam; carb, carbapenems; cef, cefazolin; chl, chloramphenicol; cip, ciprofloxacin; gent, gentamycin; imi, imipenem; lev, levofloxacin; mer, meropenem; rif, rifampin; tet, tetracycline; trim, trimethoprim-sulfate; tob, tobramycin*.

### Compound library

All compounds tested in this study are from commercially available libraries acquired and maintained in 384-well plates in DMSO at −20°C at a concentration of 2 mM by the Center for Chemical Genomics at the University of Michigan. The five libraries include MS2400, NCC, Pathways, Prestwick, and LOPAC. MS2400 is a collection of FDA approved drugs plus compounds with known biological activity obtained from Microscource Discovery (Spectrum Collection). NCC is a library with compounds that have been used in human clinical trials. The Pathways collection is comprised of known active compounds with a variety of targets. The Prestwick library is composed of approved drugs which are safe for use in humans. Finally, the LOPAC collection is the Library of Pharmacologically Active Compounds from Sigma.

### Serum bactericidal assay

After cultures were grown overnight in LB, the cells were separated from supernatant by centrifugation for 10 min at 3,500 rpm. The cells were washed in PBS and diluted 1:100. Equal volumes of cells and either 100% normal human serum, heat-inactivated human serum or factor depleted sera were incubated together for 30 min at 37°C. Samples were diluted and plated on LB agar to obtain CFUs.

### High-throughput lipase assay

Overnight cultures of wild-type (WT) *A. baumannii* 17978/p*lipBA* and 17978Δ*gspD*/p*lipBA* were grown in LB broth. After growth, the cultures were centrifuged for 10 min at 3500 rpm to separate cells and supernatant. The supernatant was removed and the pellet was washed in Mueller-Hinton 2 (MH) and resuspended in the original volume. 10 μL of MH was added to each well of a 384-well Greiner 784080 plate. Compounds in DMSO or the DMSO control were added to the wells (0.05 μL) using Perkin Elmer Sciclone liquid handler with a 50 nl pintool attachment (in primary assay). For concentration-response assays, the TTP LabTech Mosquito X1 was used to place variable volumes (0.02–1.2 μL) of compounds to the wells. 17978/p*lipBA* was diluted and added to all the negative control wells as well as to the sample wells while 17978Δ*gspD*/p*lipBA* was diluted and added to the positive control wells. All cultures had a starting OD_600_ of 0.005 and were supplemented with 50 μM IPTG (isopropyl-β-D-thiogalactopyranoside) to induce expression of the *lipBA* genes. Plates were centrifuged 1 min at 1,000 rpm to ensure all liquid was at the bottom of the well. Plates were incubated overnight at 24°C in a humidified incubator. After 16 h incubation, the OD_600_ value of each culture was recorded. An optimized lipase assay was used to measure lipase activity. Briefly, the cultures were incubated with 0.45 mM 4-nitrophenyl myristate, 80 mM Tris-HCl (pH 8.0), and 0.15% Triton X-100 and the absorbance at 415 nm was measured over time using the PE EnVision Multimode Plate Reader. All data were uploaded to MScreen for analysis. MScreen is a data analysis and storage system created by the Center for Chemical Genomics intended for the processing of high-throughput data generated by users of the center (Jacob et al., 2012).

### Lipase inhibitor assay

Cultures of *A. baumannii* 17978/p*lipBA* and 17978Δ*gspD*/p*lipBA* were grown overnight in LB broth with 50 μM IPTG and the supernatant and cells were separated by centrifugation. For concentration-response assays, the TTP LabTech Mosquito X1 was used to add variable volumes (0.02–1.2 μl) of compounds. Supernatant was added to the wells, buffer with lipase substrate was added and the change in OD_415_ was recorded as above. All data were uploaded to MScreen for analysis.

### GFP expression assay

*A. baumannii* 17978/p and 17978/p*gfp* cultures were grown O/N as above. Cells were washed as described above for the lipase assay. Compounds in DMSO or the DMSO control were added to the wells of a black low-volume Greiner 784073 plate using Perkin Elmer Sciclone liquid handler with a 50 nl pintool attachment calibrated to deliver 200 nM. For concentration-response assays, the TTP LabTech Mosquito X1 was used to place variable volumes (0.02–1.2 μl). Cultures were diluted to OD_600_ = 0.005 in LB supplemented with 75 μM IPTG. Plates were centrifuged for 1 min at 1,000 rpm to ensure all liquid was at the bottom of the well and incubated overnight at room temperature in a humidified incubator. Fluorescence was measured after growth using a BMGLabtech PHERAstar (485 nm excitation, 520 nm emission). All data were uploaded to MScreen.

### Lipase assay

A modified version of the lipase assay reported by Johnson et al. (2016) was used. Briefly, overnight cultures of *A. baumannii* strains were cultured in LB broth. The lipase activity of each culture was measured by a spectrophotometer after addition of 0.9 mM of the substrate, 4-nitrophenyl myristate in 80 mM Tris/0.15% Triton X-100 buffer. The absorbance at 415 nm was measured over time at 37°C. All assays were performed in triplicate with means and standard deviations presented.

## Results

Recent studies have demonstrated the significance of the T2SS in colonization by *A. baumannii* and *A. nosocomialis* in murine models of bacteremia and pneumonia (Elhosseiny et al., 2016; Harding et al., 2016; Johnson et al., 2016). In our study, we also revealed that one of the secreted proteins, LipA, contributes to colonization (Johnson et al., 2016), possibly by aiding in nutrient acquisition through lipid hydrolysis. It is quite likely that other T2S substrates including the lipase LipH, the phospholipase LipAN, and/or proteases and other putative enzymes identified by proteomics similarly contribute to *in vivo* survival of *A. baumannii* (Elhosseiny et al., 2016; Harding et al., 2016).

**Figure 2:**
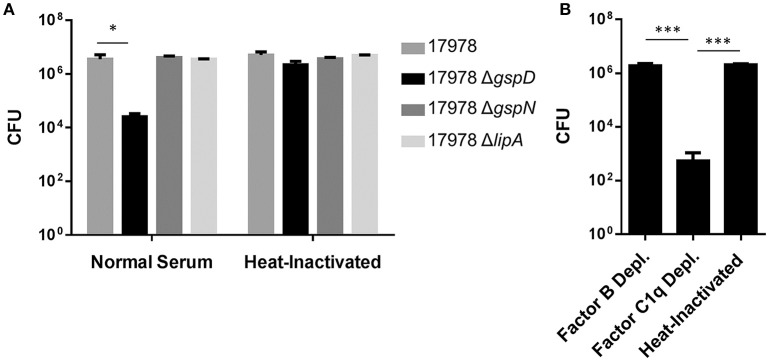
Survival in serum depends on the T2SS. **(A)** Cells from overnight cultures were washed and incubated for 30 min at 37°C with either 50% normal human serum or heat-inactivated serum. Cells were plated for CFUs following incubation. ^*^p < 0.05 by Student *t*-test; *n* = 3. **(B)** Cells from overnight cultures were washed and incubated as above with Factor B deficient, C1q deficient, or heat-inactivated serum. *n* = 3, ^***^p < 0.001 by Student *t*-test.

In addition, a “serum resistance factor” may be secreted by the T2SS, as a previous study aimed at identifying factors that contribute to *A. baumannii* proliferation in human serum identified a *gspN* transposon insertion mutant with diminished serum survival (Jacobs et al., 2010). Here, we followed up on this finding and tested the possibility that an intact T2SS is required for *A. baumannii* ATCC 17978 to resist serum complement. Many isolates of *A. baumannii* survive in the presence of 100% serum; however, ATCC 17978 is sensitive to this concentration and, therefore, we conducted our experiments using 50% serum. The WT and Δ*gspD* mutant strains were incubated in 50% pooled human sera, and the CFUs were determined after 30 min incubation at 37°C. As a control, we used the Δ*lipA* mutant that has an intact T2SS but lacks one of the T2S substrates, LipA. We also treated the WT and mutant strains with heat-inactivated (HI; 56°C, 30 min) serum, in which the complement system is inactivated. While no loss of viability was observed for the WT and Δ*lipA* strains, only 1% of the Δ*gspD* mutant cells survived 30 min in normal serum (Figure 2A). Next, we subjected the Δ*gspD* mutant cells to factor C1q-depleted and factor B-depleted human sera. The majority of Δ*gspD* mutant cells survived in the absence of factor B, which is required for activation of the alternative complement pathway; while in the C1q-depleted serum, which is deficient in the classical pathway yet contains factor B, < 0.05% of the Δ*gspD* mutant cells were viable (Figure 2B). This result suggests that the T2SS directs the outer membrane translocation of a factor that provides protection from the alternative pathway. In contrast, the Δ*gspN* mutant was not affected by human serum (Figure 2A), a result that differs from the study published by Jacobs et al. (2010). The lack of a serum sensitive phenotype for the Δ*gspN* mutant is consistent with our earlier finding that GspN is not required for T2S in *A. baumannii* and with the discovery by Wang et al. that showed GspN is not needed for survival of *A. baumannii* in a mouse model of pneumonia (Wang et al., 2014). We suggest, therefore, that the diminished growth observed for the *gspN* transposon mutant in human serum is due to a polar effect of the transposon on the downstream gene, *gspD*, which we show here is required for full protection from serum complement. Taken together with earlier findings, these results support the model that extracellular proteins secreted by the T2SS play important roles in the pathogenesis of *A. baumannii* and suggest that the T2SS may be an attractive target for therapeutic intervention.

Inhibiting the function of the T2SS would simultaneously prevent the secretion of many substrates causing a greater impact on *A. baumannii* survival than targeting individual T2S substrates. The T2SS components and their interactions (Figure 1) provide ideal targets for therapeutics because they are unique to the T2SS and are absent from most members of the human microbiota. Compounds could block the function of the outer membrane pore, inhibit interactions between the different components of the secretion apparatus, prevent recognition of the substrates by the apparatus, or interfere with the expression of the T2S proteins (Figure 1). To identify inhibitors of the T2SS, we developed a novel HTS approach.

### Development of HTS assay

**Figure 3:**
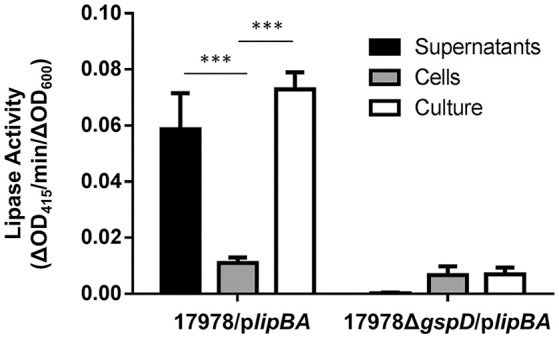
Detection of T2S lipase activity in either culture supernatants, cells, or unfractionated bacterial cultures. Enzymatic activity of either the supernatants, cells, or unfractionated cultures of 17978/p*lipBA* or 17978Δ*gspD*/p*lipBA* strains cultured overnight in LB broth. The substrate 4-nitrophenyl-myristate was added to the samples and the change in OD_415_ was recorded over time. Activities were normalized to the OD_600_ of the original cultures. *n* = 3, ^***^p < 0.001 by One-Way Anova.

In our previous study, we used a colorimetric lipase assay to measure the activity of the T2S lipase, LipA, in culture supernatant of strains overexpressing plasmid encoded *lipBA* genes. We used an overexpression strain because endogenous lipase production is very low during growth of *A. baumannii* ATCC 17978 in LB presenting a challenge for detection (Johnson et al., 2016). Further, because lipase activity is undetectable in the culture supernatant of T2SS mutants, this provides a robust assay that can be used as a readout for T2SS activity (Johnson et al., 2016) for the purpose of identifying T2SS inhibitors. However, testing the effect of a large number of compounds on LipA activity in cell-free culture supernatants would be cumbersome as it would involve an extra processing step. Thus, we compared cell-free culture supernatants and unfractionated cultures for T2SS activity (Figure 3). The culture supernatant and unfractionated culture of the 17978/p*lipBA* strain both showed significant lipase activity toward 4-nitrophenyl myristate, while there was little to no activity either in the supernatant or culture of the T2SS mutant, 17978Δ*gspD*/p*lipBA*. More importantly, the vast majority of the lipase activity in the unfractionated culture was generated by extracellular LipA thus allowing us to omit the step in which the culture supernatant is separated from cells. Consequently, we were able to develop an assay for HTS of small molecule inhibitors of T2SS that involved the addition of the lipase substrate directly to the bacterial cultures following growth in the presence and absence of compounds.

In our first attempt to miniaturize the assay we grew cultures of the WT 17978 strain and Δ*gspD* mutant in the wells of clear 96-well microtiter plates to which we then added 4-nitrophenyl myristate and determined the lipase activity by measuring the increase in absorbance at 415 nm over time. Prior to adding the lipase substrate, we measured the density of the cultures at 600 nm. This is an important step because many compounds, including known antibiotics, will affect the growth of the bacteria resulting in false positives. While the move to the microtiter format required titration of IPTG to induce *lipBA* expression, a reproducible difference in lipase activity could be measured in the WT and mutant cultures. We further developed the lipase secretion assay in flat-bottom 384-well plates using a MicromultiDrop liquid dispenser. As the conditions developed for the 96-well plates did not generate reproducible data in the 384-well format, we set up a systematic analysis in which we evaluated a variety of conditions to obtain the most consistent data. We varied growth media, culture volume, IPTG concentration, starting OD_600_ of the culture, growth temperature, length of growth, aeration, and finally, 4-nitrophenyl myristate concentration (Table 2). The experimental setup that yielded the most consistent growth and reproducible lipase activity from well-to-well and plate-to-plate were obtained when cultures were grown in 10 μL MH broth (from a starting OD_600_ of 0.005) with 50 μM IPTG, in a humidified chamber at 24°C without shaking for 16 h (Table 2).

**Table 2:** Optimization of assay conditions for development of a HTS.

**Variables**	**Conditions tested**	**Final assay condition**
Plate format	96 well vs. 384 well	384 well
Start OD	0.005, 0.0025, 0.001, 0.0005	0.005
Media	LB vs. Mueller-Hinton (MH)	MH
Volume	10, 15, 20, or 30 μL	10 μL
Temperature	20°, 24° (RT), 30°, 37°C	RT
Aeration	Shaking vs. non-shaking	Non-shaking
Substrate Concentration	0.225, 0.45, 0.9, 1.8 mM	0.45 mM

### Pilot screen

**Figure 4:**
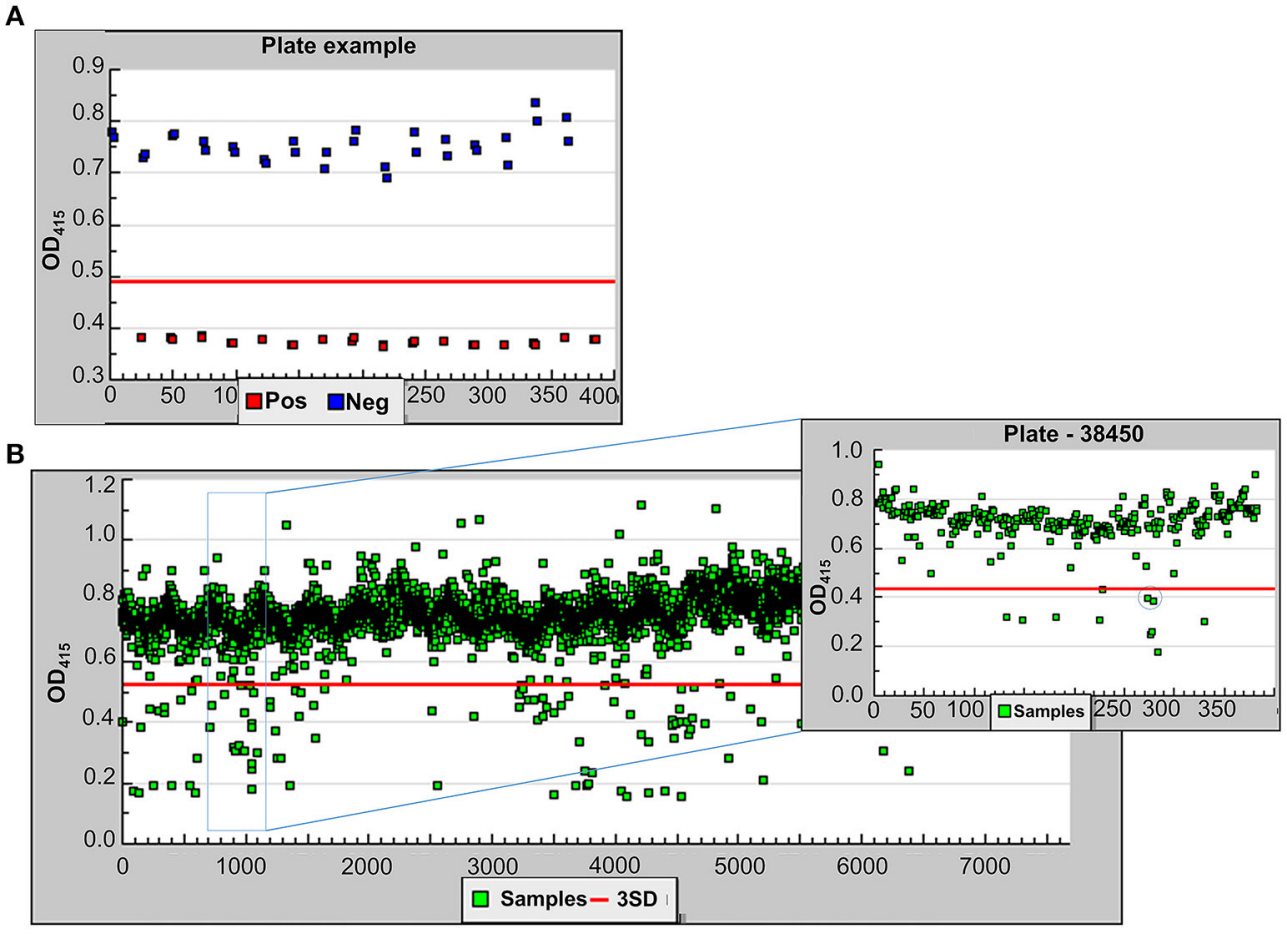
Demonstrating the feasibility of the lipase assay for HTS. **(A)** One sample plate from the pilot screen showing the OD_415_ values for the positive (red) and negative (blue) controls. The z-factor for the entire pilot screen was calculated using 1−(3^*^(σ_p_ + σ_n_)/(|μ_p_-μ_n_|)) where σ is the standard deviation and μ is the mean. **(B)** The results for 6,400 compounds tested in the pilot screen. All samples below the red line (3 *SD* from negative controls) were taken into consideration. A single sample plate is highlighted in the inset. The compound, Orlistat, indicated by the circle represents a hit in the primary screen and was tested further.

Following optimization, we screened 6,400 pharmacologically active compounds as well as FDA-approved drugs from the following libraries available at the University of Michigan Center for Chemical Genomics: MS2400, Prestwick, LOPAC, BioFocus NCC, and Focused Collections. This pilot screen was performed to evaluate the strength of the assay before moving on to larger compound libraries. Each 384-well plate contained 320 sample wells, 32 positive control wells, and 32 negative control wells. As the ultimate goal of the HTS is to identify T2SS inhibitors, the T2SS mutant, 17978Δ*gspD*/p*lipBA* served as our positive control while 17978/p*lipBA* served as the negative control. Both negative and positive controls were cultured in the presence of 0.5% DMSO while the sample wells containing 17978/p*lipBA* received the compounds resuspended in DMSO yielding a 0.5% final DMSO concentration. Following growth, the absorbance at 600 nm was measured for the cultures in each well. The average OD_600_ for the negative and positive controls were 0.21 ± 0.02 and 0.19 ± 0.02, respectively. The lipase substrate was then added, and the absorbance at 415 nm was measured over a period of 20 min at ambient temperature. The pilot screen yielded a z-factor of 0.65 [z′ = 1−(3^*^(σ_p_ + σ_n_)/(|μ_p_−μ_n_|))](Zhang et al., 1999; Figure 4A) and coefficient of variation (CV, CV = σ/μ) of 0.03 and 0.07 for the negative and positive controls, respectively. Initial active compounds were identified using statistical comparisons to positive and negative controls present on every plate. In the triage process, we selected compounds that resulted in all of the following: a reduction in lipase activity that was equal or >3 *SD* of the negative control, a minimum cut-off at 30% inhibition of lipase activity and an OD_600_ value >0.17 (Figure 4). Implementing these criteria yielded 191 compounds (3% hit rate). From these, we removed 22 compounds that are known antibiotics, such as gatifloxacin, clarithromycin, and levofloxacin and retested the remaining 169 compounds.

**Figure 5:**
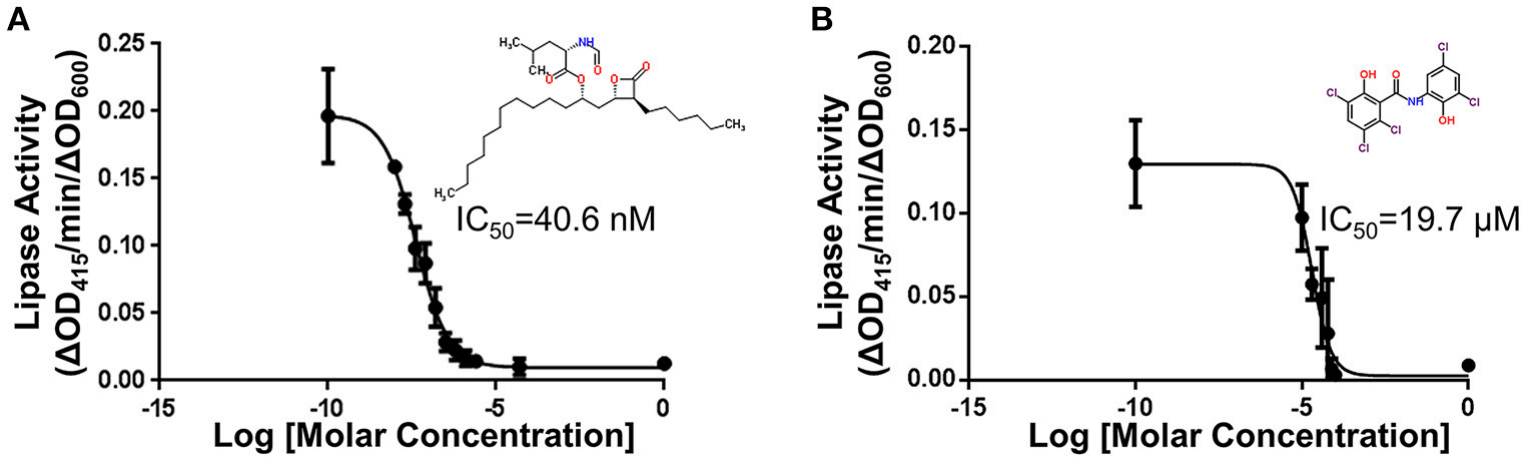
Titrations of compounds and the corresponding IC_50_. **(A)** Titration of Orlistat, a pancreatic lipase inhibitor that was identified in the primary screen. Increasing amounts of Orlistat was included during growth of 17978/p*lipBA*. After overnight incubation in 96-well plates, the substrate 4-nitrophenyl myristate was added and the lipase activity was measured. Activities have been normalized to OD_600_ of the cultures. *n* = 3, bars represent standard deviation from the mean. In follow-up counter screening, Orlistat was classified as an inhibitor of LipA activity **(B)** Titration of Oxyclozanide, an anthelmintic that was identified in the primary screen. Lipase activity in the presence of Oxyclozanide was measured as above. Activities have been normalized to OD_600_ of culture. *n* = 3, bars represent standard deviation from the mean. Following counter screening, Oxyclozanide was classified as an inhibitor of *lipA* expression from the plasmid.

Each compound was re-tested twice in a concentration-dependent manner using the original DMSO stock and covering two orders of magnitude. IC_50_ values were calculated. Forty-eight compounds gave IC_50_ values of < 30 μM. Following removal of compounds that affected growth, 34 active compounds remained (0.5% hit rate). Fresh powder of these compounds were ordered and retested. Of these compounds, 21 were confirmed as active. The compounds with the lowest IC_50_ values are known lipase inhibitors, Orlistat and Ebelactone B, and therefore likely had a direct effect on the lipase activity itself. Orlistat, a pancreatic lipase inhibitor, was the most potent of the compounds tested with an IC_50_ of 40.6 nM (Figure 5A). The other compounds exhibited IC_50_s between 4.3 and 27 μM (Table 3). The titration curves of Orlistat (Figure 5A) and Oxyclozanide (Figure 5B) are shown as examples. Of note, compounds with low IC_50_ values included tricyclic antidepressants that are known to act as serotonin-norepinephrine re-uptake inhibitors. While these latter compounds may act on the secretion system, it is possible they also bind directly to the lipase via their hydrophobic rings. Our proof-of-concept pilot screen with z' = 0.65 and CV of 0.03 and 0.07 for the negative and positive controls, respectively, showed that our assay was reproducible and was capable of identifying compounds that result in a statistically significant reduction in lipase activity. However, the identification of compounds that are known lipase inhibitors emphasized the importance of developing counter screens and other follow-up assays to remove false positives and to assure specificity in order to identify T2S inhibitors.

**Table 3:** IC_50_ values of compounds identified in primary screen and tested for concentration dependent inhibition.

**Compound**	**IC_50_ (μM)**
**LIPASE INHIBITORS**	
Ebelactone B (*n* = 2)	4.3
**ANTI-DEPRESSANTS**	
Vivactil	10
Fluoxetine	10.5
Aventyl	14.5
Duloxetine	16
Norcyclobenzaprine	16
Maprotiline	16
Desiprimine	16.5
Lofepramine	21
Sertraline	23.5
**OTHER**	
Perhexiline Maleate	4.7
Febuxostat	8
Alfuzosin	11
Stattic	12.5
Fendiline	13
Tomoxetine	14.5
Indatraline	27

### Counter screening

As the most potent compounds identified in our pilot screen described above represented lipase inhibitors, we developed a screen to eliminate these types of compounds. In this counter screen the bacterial cultures were not grown in the presence of compounds. Instead, a large batch of 17978p/*lipBA* culture was grown without compounds and following removal of cells, the cell-free culture supernatant containing the lipase was distributed in 384-well plates and incubated with the compounds, thus allowing us to identify compounds that inhibit the lipase itself. Following optimization, we identified the following conditions for the counter screen: (1) grow 17978p/*lipBA* with 50 μM IPTG for 16 h and remove cells by centrifugation; (2) dilute the supernatant 1:10 and add 10 μL to 384-well plates containing compounds; (3) add 10 μL 4-nitrophenyl myristate at 0.45 mM, incubate 10 min, and measure the change in absorbance at 415 nm.

**Figure 6:**
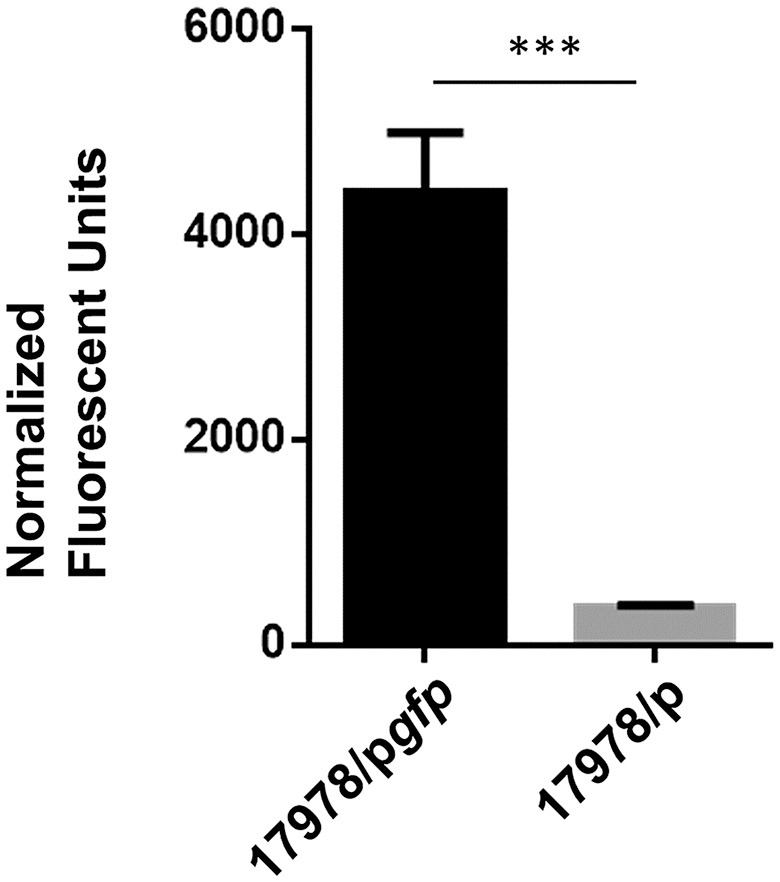
Detection of GFP fluorescence. Strains of 17978/p and 17978/p*gfp* were grown overnight in the presence of IPTG to induce the expression of GFP. After growth, fluorescence of intact cells of each strain was measured at 485 nm excitation, 520 nm emission and normalized to the OD_600_ of the cultures. *n* = 3, ^***^p < 0.001 by Student *t*-test.

An additional counter screen was developed for the elimination of compounds that interfere with plasmid-encoded *lipBA* expression. For this, we used the same plasmid backbone as p*lipBA* but substituted the *lipBA* gene with the *gfp* gene, which codes for Green Fluorescent Protein (p*gfp*). This plasmid was introduced into the WT 17978 strain (17978/p*gfp*), and without lysing the cells, reproducible GFP fluorescence was significantly higher than the fluorescence detected for 17978 with the vector alone negative control (17978/p; Figure 6). We optimized the conditions and applied them in the following counter screen: cultures were grown in 10 μL MH from a starting OD_600_ of 0.005, with 75 μM IPTG in a humid chamber at 24°C without shaking for 16 h using 17978/p*gfp* and 17978/p as negative and positive controls, respectively. For this counter screen, the compounds would be added to the cultures at the start of growth, with the fluorescence measured after growth. Any compound that is positive in both the primary screen and this counter screen is likely targeting expression of the plasmid-encoded lipase and should be removed from the pool of potential hits.

Twenty-one compounds that had responded in a concentration-dependent manner in the primary HTS and then confirmed when using fresh powders, were subjected to the lipase inhibitor and GFP counter screens. All of these compounds were identified as either lipase inhibitors (*n* = 18; Table 3 and Figure 5A) or inhibitors of *lipBA* expression from the plasmid (*n* = 3; representative compound shown in Figure 5B) highlighting the necessity of utilizing counter screens before pursuing detailed characterization of false positives.

**Figure 7:**
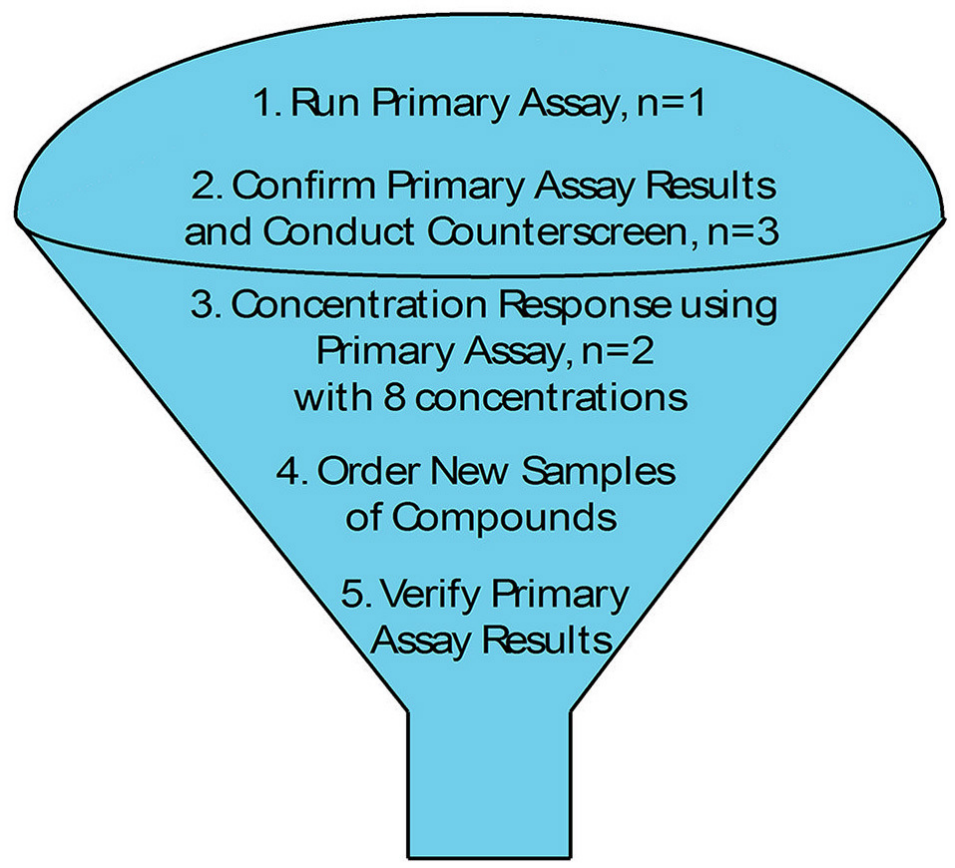
HTS Schematic.

**Figure 8:**
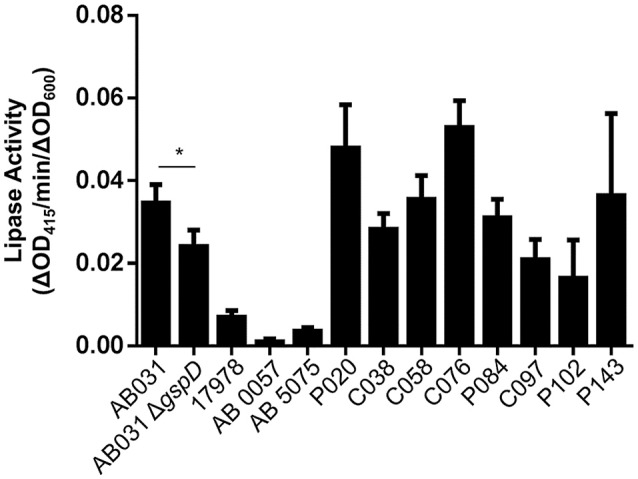
T2S lipase activity of *A. baumannii* strains. The lipase substrate, 4-nitrophenyl myristate was added to overnight cultures of the indicated *A. baumannii* strains and the lipase activity was measured. Activities were normalized to the OD_600_ readings of each culture. Bars show standard deviations from the means, *n* = 3. ^*^p < 0.05 by Student *t*-test.

As we move forward to screen larger libraries to identify T2SS inhibitors, our protocol will involve the following order of analysis (Figure 7): the active compounds from the primary HTS (step 1) will be delivered in triplicates to three different sets of 384-well plates using the original DMSO stock solutions (step 2). The first set of plates will represent a repetition of the primary screen. The second and third sets of plates will be used to counter screen for compounds that inhibit lipase activity or plasmid-borne gene expression, respectively (Figure 7). Compounds that are positive in the counter screens will be eliminated from further consideration, and the remaining compounds will be tested for their ability to prevent secretion over a range of concentrations (step 3). Fresh compounds will be reordered and tested (steps 4 and 5). Active compounds will then be analyzed for inhibition of secretion in additional *A. baumannii* strains, as T2SS inhibitors should ideally be functional against the T2SS of all the *A. baumannii* isolates regardless of antibiotic resistance phenotype. To this effect, we have begun to test lipase activity of other strains of *A. baumannii* that were isolated from different body sites, are resistant to different antibiotics and produce different amounts of biofilm (Figure 8). While we have previously shown that detection of lipase activity in the culture supernatant of ATCC 17978 grown in LB in the absence of lipids requires overexpression of LipA from a plasmid, other strains display lipase activity without the need for LipA overexpression (Figure 8). The difference in lipase activity among the strains may be due to differences in expression of *lipA* as well as the presence of other lipases, such as LipH, which may or may not be dependent on the T2SS for extracellular secretion. We constructed a T2SS mutant of one of the strains, AB031 (to be described elsewhere). This Δ*gspD* mutant had a statistically significant reduction in lipase activity compared to the WT AB031 strain indicating that a detectable portion of the lipase activity stems from a T2SS dependent lipase(s) (Figure 8). This strain, as well as any others we may find as we continue to screen *A. baumannii* isolates for extracellular lipase activity, may be used to further analyze active hits to help determine which compounds to pursue. Analysis of additional strains such as AB0057 and AB5075 that show low lipase activity, however, will likely involve plasmid-expression of LipA.

## Discussion

Here, we provide additional information on the T2SS in *A. baumannii*. In addition to supporting colonization, in part through the secretion of LipA, we show that the T2SS also contributes to serum resistance as there is a ≥100-fold reduction in recoverable CFUs of ATCC 17978 Δ*gspD* mutant following exposure to human complement. The mechanism by which the T2SS protects *A. baumannii* from human complement is not known, but published reports have shown that *A. baumannii* expresses secreted and surface-associated proteins that contribute to serum resistance and it is possible that they use the T2SS for outer membrane translocation. The serine protease PKF is produced with an N-terminal signal peptide, a prerequisite for T2S, and CipA, another serum resistance factor is a predicted lipoprotein (King et al., 2013; Koenigs et al., 2016). Lipoproteins, such as pullulanase and SslE, are examples of surface-associated T2S substrates, and CipA may similarly localize to the cell surface via the T2SS (Pugsley et al., 1986; Baldi et al., 2012). While PKF is a member of the HtrA family of chaperone-proteases that refold or degrade misfolded proteins in the periplasmic compartment (Hansen and Hilgenfeld, 2013), it is detected in *A. baumannii* culture supernatants possibly due to its secretion via the T2SS.

The reduced *in vivo* fitness in mouse models of bacteremia (Johnson et al., 2016) and pneumonia (Elhosseiny et al., 2016) and the increased sensitivity to complement killing of T2SS deficient *A. baumannii* (Figure 2) suggest that the T2SS plays an important part during infection through the action of specific T2S substrates and, thus, shows great potential for therapeutic targeting. GspD may be an especially promising target, as it forms a gated channel in the outer membrane through which transport occurs. Potential drugs, therefore, would not need to penetrate membranes to reach their target and would not be subject to the effect of drug efflux pumps. This latter point may be of particular importance in the treatment of bacteremic *A. baumannii*, as the expression of several efflux pumps are upregulated when *A. baumannii* is cultured in human serum (Jacobs et al., 2012). Here, we describe the development and optimization of a HTS to identify small molecule inhibitors of the T2SS in *A. baumannii*. Using a previously published assay, we developed, optimized, and tested a high-throughput assay on a small library of pharmacologically active compounds. Our proof-of-concept study demonstrated little fluctuations within controls and showed an acceptable dynamic range between positive and negative controls yielding a z-factor of 0.65 (Zhang et al., 1999). It also indicated that the assay is simple, straightforward, reproducible, robust, and specific as it identified the lipase inhibitors Orlistat and Ebelactone B twice and all seven tricyclic antidepressants present among the 6,400 compounds tested. In addition, the pilot study underscored the importance of including counter screens to reduce the number of false-positives. Though we are confident that our primary lipase screen and counter screens have been sufficiently optimized to be used to screen larger libraries of compounds, we may add an additional assay to reduce the number of potential hits early in the course of triage. If active compounds identified in the HTS are true inhibitors of the T2SS they should also block the secretion of other T2S substrates. Therefore, we may consider employing a double screen that simultaneously detects a reduction of the extracellular amount of LipA and another T2S enzyme. This assay has yet to be developed; however, mass spectrometry analysis of culture supernatants of *A. baumannii* and the related *A. nosocomialis* indicate that several Acinetobacter enzymes besides LipA are secreted by the T2SS (Elhosseiny et al., 2016; Harding et al., 2016), suggesting that a double screen is feasible.

Once larger libraries are subjected to this HTS and the primary assay results have been verified with fresh powders (Figure 7), the high-purity compounds will be further tested for specificity. While inhibitors that target the essential Sec system and signal peptidase (Tsirigotaki et al., 2017) would be detected and removed in the primary screen due to their negative impact on growth, a partial growth defect may not be observed in our end point measurement of cell density. This will be addressed in two ways. First, cultures will be grown in microtiter plates in the absence or presence of verified compounds and the OD_600_ will be measured over time. Second, the activity of a periplasmic enzyme, such as alkaline phosphatase, will be determined. This activity will be reduced when the activity of the Sec system is diminished. Other compounds may target periplasmic chaperones or disulfide isomerases such as DsbA (Goemans et al., 2014). The alkaline phosphatase assay will also be instrumental in the detection and subsequent removal of these compounds.

Other studies have implemented similar HTS approaches for the identification of secretion inhibitors with notable differences. The first screen developed and validated, used a gain-of-signal screen to identify inhibitors of SecA, an essential component of the Sec export pathway in *P. aeruginosa* (Moir et al., 2011). No inhibitors were identified to directly interfere with the Sec pathway as the transport of the periplasmic enzyme β-lactamase was not affected. However, following application of secondary assays, a set of compounds was found to reduce the extracellular activity of T2SS substrates although they had no effect on the secreted substrates themselves, suggesting that the compounds inhibit their secretion (Moir et al., 2011). Our screen differs from the Sec screen in that we use a T2SS mutant as our positive control, thus increasing the specificity of our assay. In addition, our screen is designed to include high-throughput counter screens for the removal of false positives early in the triage process. The screen developed and validated by Tran et al., utilized the plant pathogen *Dickeya dadantii* (Tran et al., 2013). Similar to our screen, the authors measured OD_600_ after growth to detect antibiotics and used the activity of a T2S substrate to measure the functionality of the T2SS. As with the Moir et al., screen, the Tran et al., screen did not have counter screens developed to be utilized in the HTS process (Moir et al., 2011; Tran et al., 2013). What is apparent in all three studies is that identified compounds have to be subjected to many additional tests, including those mentioned above, before they can be classified as true T2SS inhibitors. In addition, subcellular fractionation of cells grown in the presence of active T2S inhibitors should confirm an accumulation of T2S substrates in the periplasmic compartment, although some T2S substrates may be degraded by periplasmic proteases when their outer membrane translocation is perturbed. Once we implement our screen for the identification of T2SS inhibitors in *A. baumannii*, it will be advantageous to compare any active compounds to the compounds discovered in these two screens and search for similarities amongst the compounds.

While the primary goal of this pilot screen was to develop a HTS regimen for the identification of compounds to target the T2SS, our data indicate that our dual screen combined with counter screens also have the potential to identify compounds with antibiotic properties and reveal new targets for known pharmacological compounds already in use. An example of this is our discovery that the tricyclic antidepressants are efficient inhibitors of *A. baumannii* LipA (Table 3). Less surprising was the finding that the pancreatic lipase inhibitors Orlistat and Ebelactone B efficiently inhibit LipA activity. However, as our previous study has shown that LipA enhances *A. baumannii* colonization (Johnson et al., 2016) and that *A. baumannii* secretes several lipolytic enzymes including LipH and LipAN that may also support *in vivo* survival of *A. baumannii* (Elhosseiny et al., 2016; Harding et al., 2016), a lipase inhibitor could have potential for therapeutic use. Along with T2SS inhibitors, our HTS may also isolate inhibitors of LipB, the chaperone for both LipA and another T2S substrate, LipH (Harding et al., 2016), and they may also be developed for therapeutic intervention.

Identification of T2SS inhibitors for therapeutic purposes is the ultimate goal; however, we are also interested in pursuing small molecules, which can be used as tools to study T2S in multidrug resistant strains as they are often genetically intractable and very difficult to systematically study. Therefore, development of chemical probes to advance virulence studies of these antibiotic resistant isolates is also critically important.

## Author contributions

MS, UW, HM, and LS conceived the experiments. CX provided clinical strains. UW, TJ, and KC performed the experiments. UW and MS analyzed the data and wrote the manuscript. All authors have read and approved the manuscript.

### Conflict of interest statement

The authors declare that the research was conducted in the absence of any commercial or financial relationships that could be construed as a potential conflict of interest.
